# Progress of Surgery

**Published:** 1904-01-30

**Authors:** 


### Progress of Surgery.

(*Continued from Page* 294.)

Cystoscopy.—F. Cabot and H. G. Spooner (New York)[2] have written a paper on the cystoscope and what can be expected and what cannot be expected from cystoscopic exploration in genito-urinary diseases. Three conditions are necessary for successful cystoscopic examination, viz., (1) the urethra must be large enough to admit a sound of 24 calibre of the French scale. (2) The bladder must tolerate the presence of 90 to 120 grammes of fluid at least. (3) The wall of the bladder must remain transparent during the examination. Enlargement of the prostate may interfere with cystoscopy. The authors usually introduce 120 gm. of boric acid solution (= ℥ v). Ten or 15 centigrams (= about ʒ iii) of a 10 per cent. solution of cocaine can be injected into the bladder without great danger. In cases of hæmorrhage from the bladder adrenalin chloride, 1 in 5,000, is an effective remedy. The authors describe the technique of cystoscopy and explain the reversal of the images produced. When a bladder tumour with villi is finely pedunculated the chances are that it is not malignant. When the villosities jut out from a solid mass, epithelioma should be thought of. Stones in the bladder have been revealed by the cystoscope which the sound had failed to detect. Ulcerations of the bladder were rare, but the cystoscope alone revealed their presence.

The Surgical Treatment of Chronic Bright's Disease.—Ramón Guiteras has discussed this question.[3] He was still uncertain whether it was better to partially decapsulate the kidneys and fix them, or to entirely decapsulate and replace them in their fatty capsules. He had begun these operations in the previous year as a sceptic, but his experience and the growing favour which leading surgeons were giving the operation led him to send a circular letter to surgeons to ascertain their experience. It appeared from the 150 answers he had received that surgeons in favour of and those against the operation were about equally divided. The theory was that the circulation in the kidney was increased by anastomosis with the surrounding tissues. The conditions considered favourable for operation were movable kidney with casts and albumin, or associated with nephritis, chronic interstitial nephritis, and diffuse nephritis. Over 100 cases of the operation had been reported, 16 per cent. as cured, 40 per cent. as improved, 11 per cent. as unimproved, and 33 per cent. as fatal. The cases of chronic diffuse nephritis were the most favourable for operation. Many of the patients operated on had been suffering from general anasarca, nearly all had uræmic symptoms, and 75 per cent. of them had been œdematous. Movable kidney was often associated with albuminuria and cylindruria. This was not genuine Bright's disease, and could be cured by fixing the kidney. It was generally acknowledged that true Bright's disease was always bi-lateral. He had looked over the records of 500 autopsies in cases of chronic Bright's disease, and in all of these the disease had been bi-lateral, and in only 14 had one organ been more involved than the other. He did not believe that it was necessary to entirely decapsulate the organ to afford relief. He felt that a partial method of decapsulation with fixation might prove to be the better by reason of the scanty vascularity of the fatty capsule. Experiments tended to show that the blood supply obtained from the fatty capsule was very meagre after decapsulation. After decapsulation a fibrous investment developed often thicker than the original one, which replaced the normal capsule. For patients with uræmic symptoms or chronic œdema, an operation could be resorted to in case medical treatment had proved of no avail. In cases of anasarca, especially if the heart was very bad, it was a question whether one should operate or not. The reports showed 50 per cent. mortality in such cases, 25 per cent. were unimproved, and 25 per cent. were improved or cured. He had never had the courage to operate in these cases Dr. G. M. Edebohls said it would be from two to five years before it would be known exactly what these operations could do for Bright's disease. Out of 59 of his patients operated on 11 could be safely considered to have been permanently cured. A patient should be free from albumin and casts for a period of six months at least before he could be considered cured. A certain amount of improvement after operation had been almost uniform. He would not anchor such kidneys unless the patient presented symptoms due to mobility. He had examimed post-mortem two kidneys which had been operated on four months previously and had found enormously dilated and enlarged blood-vessels penetrating from the fatty capsule through the capsule proper into the kidney substance, the capsule proper having been reproduced.

Injuries of the Kidneys.—F. M. Watson[4] has contributed a paper on this subject based on a collection of 660 cases. The kidney might be ruptured by muscular action, and of this he had found 10 instances. In some, very inadequate force seemed to have produced the accident. Blows or falls on the front of the abdomen were not infrequently the cause. If other organs were injured at the same time, these were nearly always on the same side. Peritoneal irritation, as shown by vomiting, tympanites, and muscular rigidity, was often absent. Hæmaturia in several cases was delayed for from 1 to 7 days. It had been absent in 7 out of 340 cases. Its absence might be due to tearing across of the pelvis, ureter, or renal vessels, complete disintegration of the kidney, thrombosis of the blood-vessels, and obstruction of the ureter by blood-clot. It might persist for a long time without fatal results—*e g.*, for 56 and 46 days in 2 cases. Tumour in the loin was noted in 103 out of 486 cases. The swelling was in 41 a traumatic hæmatoma; in 26 a traumatic hydronephrosis; and in 32 a perinephritic abscess. Some increase of the hæmatoma during the first 24 or even 48 hours, if gradual, was not necessarily a dangerous indication. Hæmorrhage was responsible for the largest number of deaths. The following inferences as to when operation was indicated seemed justifiable:—(1) The presence of hæmaturia severe enough to produce the signs of serious hæmorrhage; (2) the presence of a rapidly-increasing tumour in the loin; (3) the development of a tumour in the loin ten or more days after the injury; (4) immediate operation was indicated when there was evidence pointing to peritonitis or other intra-peritoneal lesion.

[2] Med. Rec., July 11, 1903, p. 57. [3] Ibid., Sept. 19, 1903, p. 474. [4] Ibid., p. 476.

*(To be continued.)*

